# Prevalence and Factors Associated with Self-Medication in Dermatology in Togo

**DOI:** 10.1155/2017/7521831

**Published:** 2017-11-12

**Authors:** Koussake Kombaté, Julienne Noude Técléssou, Bayaki Saka, Abla Sefako Akakpo, Koudjouka Odette Tchangai, Abas Mouhari-Toure, Garba Mahamadou, Waguena Gnassingbé, Aurel Abilogun-Chokki, Palokinam Pitché

**Affiliations:** ^1^Service de Dermatologie et IST, CHU Lomé, Université de Lomé, Lomé, Togo; ^2^Service de Dermatologie et IST, CHU de Kara, Université de Kara, Kara, Togo

## Abstract

**Objective:**

This study aimed to determine the prevalence of and factors associated with self-medication in dermatology in Lomé, Togo.

**Methods:**

We conducted an analytical cross-sectional study from February to April 2016 in 2 dermatology departments in Lomé. Univariate and multivariate logistic regression models were carried out to identify possible factors associated with self-medication.

**Results:**

A total of 711 patients were included in the study. The mean age (±SD) of the patients was 26.6 ± 6.9 years and the sex ratio (male/female) was 0.6. The main dermatologic diseases recorded were immunoallergic dermatoses (39.7%) and infectious skin diseases (22.6%). Two-thirds (481/711; 66.7%) of the patients had practiced self-medication before consultation in dermatology units. In multivariate analysis, factors associated with self-medication were female sex (aOR = 1.44; 95% CI = [1.01, 2.05]), duration of dermatologic disease more than one year (aOR = 1.79; IC = [1.19, 2.68]), adnexal dermatoses (aOR = 2.31; 95% IC = [1.03–5.21]), keratinization disorders (aOR = 4.23; 95% CI = [1.36–13.13]), and fungal skin infections (aOR = 5.43; 95% CI = [2.20, 13.38]).

**Conclusion:**

Our study confirms that self-medication practice is very common among patients with dermatologic diseases in Lomé and has identified associated factors.

## 1. Introduction

In sub-Saharan Africa, there is a wide range of therapeutic options, ranging from modern medicine to traditional medicine, religious cults or healing prayer, and self-medication. In this wide range, self-medication occupies an important part for social, economic, and psychological reasons [1]. The World Health Organization (WHO) defines self-medication as practices by which people deal with aches and health conditions with drugs that are approved and available without prescription and are safe and effective when used according to inscriptions [2]. In addition, self-medication is called anarchic when treating a real or imagined pathological condition by drugs selected without medical advice or without consulting a health care professional in his area of competences [2]. This includes self-prescribing but excludes drug addiction [3]. In dermatology, self-medication can be the cause of serious drug reactions [4–8]. A previous study conducted in dermatology units in Lomé, reported a prevalence of self-medication at 44% [9], but it had not identified associated factors. The aim of this study was to determine the prevalence of and factors associated with self-medication in patients with dermatologic diseases in Lomé, Togo.

## 2. Materials and Methods

### 2.1. Study Design

An analytical cross-sectional study was conducted from February to April 2016 in two dermatology units (dermatology unit of CHU Campus and dermatology center of Gbossimé) in Lomé.

### 2.2. Study Population and Sampling

During the study period, all patients aged over 15 years who consulted for dermatologic diseases for the first time and who gave their consent were included in the study. The cost of dermatological consultation in these centers was 3000 CFA (5 euros) for patients without health insurance and 600 CFA (1 euro) for patients with health insurance.

### 2.3. Data Collection

A semistructured questionnaire was used to collect data through an interview in French or in the local language, conducted by the clinic officers to ensure good understanding of the questions.

The questionnaire included sociodemographic information, health insurance status, clinical features, and history of treatments before the admission in dermatology unit. In this study, we considered medical prescription as any treatment received on medical prescription by a health professional. A self-medication was considered as all other aspects of therapeutic used by patients (self-prescription, traditional medicine, and religious prayers).

### 2.4. Data Analysis

Data entry and analysis were performed using Epi-info version 3.5.1 software. For continuous variables, mean and standard deviation were calculated while for categorical variables we calculated proportions. Pearson chi-square test was used in bivariate analysis. The significance threshold was set at 5%. Multivariate backwards stepwise logistic regression analysis was performed to identify associated factors of self-medication. All variables significant during bivariate analysis at a *p* value less than 0.05 were introduced in a logistic regression model to appreciate the adjusted effect and derive the adjusted odds ratio (aOR) of each on the primary outcome, “self-medication” expressed as a dichotomous variable. A 95% level of confidence was applied throughout.

## 3. Results

### 3.1. Characteristics of the Study Population

In total, 711 patients were included in the study during the study period, of which 439 were women. The mean age of the patients was 26.58 ± 6.9 years (range: 15–74 years). Of the 711 patients, 17% had health insurance, 79.7% had formal education, and more than two-thirds traveled less than 15 kilometers to consult a dermatologist. The main dermatologic diseases were immunoallergic dermatoses (39.7%) and infectious skin diseases (22.6%) (Table 1).

### 3.2. Medications before Admission in Dermatology

Previous therapies were reported in 529 (74.4%) of the 711 patients prior to admission to dermatology units. Of these 529 patients, 48 (8.9%) had used medical prescription only and 481 (91.1%) had used self-medication only or associated with medical prescription. In total, of the 711 participant patients, 481 (67.7%) practiced self-medication only or associated with medical prescription for their dermatologic disease before consultation in dermatology unit. The main examples of medicines used in self-medication were traditional drugs (mixed decoctions of medicinal plants and ingredients), Chinese drugs, or modern medical drugs without medical advice. In bivariate analysis, factors associated with self-medication were female sex (*p* = 0.02), education level (*p* = 0.02), distance traveled to see a dermatologist more 30 kilometers (*p* = 0.04), duration of dermatologic disease more than one year before consultation (*p* = 0.03), and types of skin diseases (*p* < 0.01) (Table 1).

In multivariate analysis, factors associated with self-medication prior to consultation in dermatology were female sex (aOR = 1.44; 95% CI = [1.01, 2.05]), duration of dermatologic disease more than one year (aOR = 1.79; 95% CI = [1.19, 2.68]), and having developed the following 3 types of skin diseases: adnexal dermatoses (aOR = 2.31; 95% IC = [1.03–5.21]), keratinization disorders (aOR = 4.23; 95% CI = [1.36–13.13]), and fungal skin infections (aOR = 5.43; 95% CI = [2.20; 13.38]). High education level was a protective factor against self-medication (aOR = 0.52; 95% CI = [0.30–0.89]) (Table 2).

## 4. Discussion

This study confirms that self-medication is widely practiced among patients with dermatologic diseases in Lomé. It identified 5 factors associated with self-medication which are female sex, duration of dermatologic disease more than one year before consultation, and having developed the following type of skin diseases: adnexal dermatoses, keratinization disorders, and fungal skin infections. High education level is found as a protective factor against self-medication. Finally, we found that having or not a health insurance does not influence the practice of self-medication.

The prevalence of self-medication we report in this study is higher than those reported previously in the same unit. Indeed, the prevalence of self-medication was 44% in patients with dermatologic diseases [9] and 47% among patients who developed Stevens-Johnson and Lyell syndromes [5]. The sincerity of information gathered during the interview with some patients having the fear or the shame of confessing to their practice of self-medication could explain the large differences. Other studies have reported prevalence of self-medication varying between 6% and 46.7% among patients with dermatologic diseases [10, 11]. The frequency of self-medication reported in our study (67.7%) is almost similar to one (71.9%) reported among patients with rheumatic diseases in Burkina Faso [12]. The pressure of the family in decision-making, the difficulties of access to a dermatology specialist and the embarrassment or shame of the disease, and especially the availability of multiple therapies outside health facilities and drugs delivery points are as many factors that encourage self-medication in sub-Saharan Africa. In dermatology, self-medication can lead to many cutaneous reactions; preventive actions and health education in the community are necessary to reduce or treat this problem.

In our study, the female sex, duration of dermatologic disease more than one year before consultation, and 3 types of skin diseases were factors associated with self-medication. The female sex was also identified in the study of Ouédraogo et al. [12] as a factor associated with self-medication in rheumatic diseases. Both studies show that the female sex is a risk factor for self-medication regardless of the specialty concerned. The likely explanation we propose is the women's easy access to illicit medicines in markets and streets often used in self-medication. Unlike the study of Ouédraogo et al. [12], high education level was a protective factor against self-medication in our study. Indeed, educated patients know the misdeeds of self-medication and therefore could avoid it. Similar findings were reported in a Mexican study where the illiterate and the low-educated subjects were those who practiced more self-medication [13].

We also identified the fungal skin infections as being associated with the risk of self-medication. Poudyal and Joshi [14] noted that self-medication for dermatophytosis was frequent and the most commonly used medicines for self-medication were topical steroids alone or associated with antifungal drugs. The chronic, recurrent, and pruritic characteristics of some dermatophytoses probably explain this fact. Finally, having chronic (duration of dermatologic disease more than one year before consultation) or persistent (adnexal dermatoses and keratinization disorders) dermatoses leads the patients to practice self-medication.

## 5. Limitations

The main limitation of this study is related to the sincerity of the information gathered during the interview, with some patients having the fear or the shame of confessing to their practice of self-medication. This could explain the large difference between the rate reported in this study and those reported previously in the same unit. Due to the cross-sectional study design of this study, we could not assess the temporality of the associated factors and the outcome.

## 6. Conclusion

Our study confirms that self-medication is widely practiced among patients with skin diseases in dermatology units in Lomé and identified associated factors which are female sex, delay of consultation more than one year, having dermatologic diseases such as fungal skin infections, adnexal dermatosis, or keratinization disorders. It is necessary for decision-makers to develop strategies to bring dermatology services closer to the population especially in remote areas. In addition strengthening health promotion among the population will improve the use of health services and reduce self-medication.

## Figures and Tables

**Table 1:** Sociodemographic and clinical characteristics of patients with dermatologic diseases practicing self-medication in Lomé, 2016.

Characteristics of patients	Total *N* = 711 (%)	Self-medication*N* = 711		*p* value
		Yes *N* (%)	No *N* (%)	
*Age*				
More than 60 years	9 (1.3)	5 (55.5)	4 (44.5)	0.40
35–60 years	193 (27.1)	140 (72.5)	53 (27.5)	
15–35 years	509 (71.6)	336 (66.0)	173 (44.0)	
*Sex*				
Female	439 (61.8)	284 (64.7)	155 (35.3)	*0.02*
Male	272 (38.2)	197 (72.4)	75 (27.6)	
*Health insurance*				
No	590 (83.0)	406 (68.8)	184 (31.2)	0.13
Yes	121 (17.0)	75 (61.9)	46 (38.1)	
*Education level*				
Nonschooled	144 (20.3)	98 (68.1)	46 (31.9)	
Primary school	180 (25.3)	136 (75.6)	44 (24.6)	*0.02*
Secondary school	241 (33.9)	161 (66.8)	80 (33.2)	
High level school	146 (20.5)	86 (58.9)	60 (41.1)	
*Distance traveled*				
Less than 15 kilometers	564 (79.3)	369 (65.4)	195 (34.6)	
15–30 kilometers	59 (8.3)	42 (71.2)	17 (28.8)	*0.04*
30–100 kilometers	62 (8.7)	49 (79.0)	13 (21.0)	
More than 100 kilometers	26 (3.7)	21 (80.8)	5 (19.2)	
*Duration of dermatologic disease before consultation*				
Less than 3 months	233 (32.8)	145 (62.2)	88 (37.8)	*0.03*
3–6 months	136 (19.1)	61 (44.9)	75 (55.1)	
6–12 months	55 (7.7)	9 (16.4)	46 (83.6)	
More than 12 months	287 (40.4)	266 (92.7)	21 (7.3)	
*Reasons for consultation*				
Immunoallergic dermatosis	282 (39.7)	194 (68.8)	88 (31.2)	*<0.01*
Tumoral dermatosis	21 (3.0)	11 (52.4)	10 (47.6)	
STI	19 (2.7)	14 (73.7)	5 (26.3)	
Adnexal dermatosis	55 (7.7)	38 (69.1)	17 (30.9)	
Bacterial skin infections	53 (7.4)	37 (69.8)	16 (30.2)	
Viral skin infections	28 (3.9)	13 (46.4)	15 (53.6)	
Fungal skin infections	70 (9.8)	61 (87.1)	9 (12.9)	
Parasitic skin infections	11 (1.5)	9 (81.8)	2 (18.2)	
Keratinisation disorders	29 (4.1)	24 (82.8)	5 (17.2)	
Dyschromia	41 (5.8)	24 (58.5)	17 (41.5)	
Dysimmunity dermatosis	46 (6.5)	26 (56.5)	20 (43.5)	
Others skin diseases	56 (7.9)	30 (53.6)	26 (46.4)	

STI: sexual transmitted infections.

**Table 2:** Factors associated with self-medication among patients with dermatologic diseases in Lomé, 2016.

Characteristics of patients	aOR	95% CI
*Female sex*	*1.44*	*[1.01; 2.05]*
*Education level*		
Nonschooled	*Ref*	
Primary school	*1.28*	[0.76; 2.15]
Secondary school	*0.89*	[0.55; 1.43]
High level school	*0.52*	[0.30; 0.89]
*Distance traveled*		
Less than 15 kilometers	*Ref*	
15–30 kilometers	*1.27*	[0.68; 2.35]
30–100 kilometers	*1.89*	[0.94; 3.78]
More than 100 kilometers	*2.13*	[0.76; 5.98]
*Duration of dermatologic disease before consultation*		
Less than 3 months	*Ref*	
3–6 months	*0.99*	[0.61; 1.59]
6–12 months	*0.90*	[0.47; 1.72]
More than 12 months	*1.79*	*[1.19; 2.68]*
*Reasons for consultation*		
Other skin diseases	*Réf*	
Immunoallergic dermatosis	*1.84*	[0.99; 3.38]
Tumoral dermatosis	*0.96*	[0.33; 2.80]
STI	*2.78*	[0.85; 9.06]
Adnexal dermatosis	*2.31*	*[1.03; 5.21]*
Bacterial skin infections	*1.94*	[0.85; 4.39]
Viral skin infections	*0.66*	[0.25; 1.72]
Fungal skin infections	*5.43*	[2.20; 13.38]
Parasitic skin infections	*3.47*	[0.64; 18.79]
Keratinisation disorders	*4.23*	*[1.36; 13.13]*
Dyschromia	*1.26*	[0.54; 2.92]
Dysimmunity dermatosis	*0.99*	[0.43; 2.25]

STI: sexual transmitted infections.

## Abbreviations

aOR: Adjusted odds ratio
STI: Sexual transmitted infections
WHO: World Health Organization.

## References

[1] Manzambi J. K., Tellier V., Bertrand F., Albert A., Reginster J. Y., Van Balen H. (2000). The determinants of recourse behavior at a health center in African urban surroundings: results of a survey in families at Kinshasa, Congo. *Tropical Medicine & International Health*.

[2] World Heart Organization. Guidelines for the regulatory assessment of medicinal products for use in self-medication. WHO/EDM/QSM/00.1, Geneva 2000

[3] Jolliet P. (2004). Self-medication. *Revue du Praticien*.

[4] Estève E., Ah-Toye C., Nseir A., Martin L.

[5] Saka B., Kombaté K., Egbohou P. (2012). Stevens-Johnson syndrome and toxic epidermal necrolysis in Togo: the role of self-medication. *Annales de Dermatologie et de Venereologie*.

[6] Adams B. B., Sheth P. B. (2002). Perianal ulcerations from topical steroid use. *Cutis; Cutaneous Medicine for the Practitioner*.

[7] Kastalli S., El Aidli S., Daghfous R. (2004). Fixed drug eruption induced by sulfaguanidine. *Annales de Dermatologie et de Venereologie*.

[8] Neema S., Chatterjee M., Mukherjee T., Jha S. (2016). Pigmented contact dermatitis resulting from self-medication for postherpetic neuralgia. *Indian Journal of Dermatology*.

[9] Mouhari-Toure A., Kombaté K., Saka B., Akakpo S., Boukari O. B. T., Pitché P.

[10] Treesirichod A., Chaithirayanon S., Chansakulporn S. (2015). Self-medication for dermatologic diseases among children treated at the HRH princess maha chakri sirindhorn medical center. *Journal of the Medical Association of Thailand*.

[11] Corrêa-Fissmer M., Martins A. H., Mendonça M. G., Galato D. (2014). Prevalence of self-medication for skin diseases: a systematic review. *Anais Brasileiros de Dermatologia*.

[12] Ouédraogo D., Zabsonré Tiendrebeogo J. W., Zongo E. (2015). Prevalence and factors associated with self-medication in rheumatology in Sub-Saharan Africa. *European Journal of Rheumatology*.

[13] Balbuena F. R., Aranda A. B., Figueras A. (2009). Self-medication in older urban Mexicans: an observational, descriptive, cross-sectional study. *Drugs & Aging*.

[14] Poudyal Y., Joshi S. D. (2016). Medication practice of patients with dermatophytosis. *Journal of Nepal Medical Association*.

